# FDA-Approved Hydrogel-Mediated In Situ Sonodynamic and Chemotherapeutic Therapy for Pancreatic Cancer

**DOI:** 10.3390/ph17121666

**Published:** 2024-12-10

**Authors:** Jian Wang, Nianhui Yu, Yunpeng Tang, Yingsheng Cheng, Hui Li

**Affiliations:** 1 Department of Radiology, Sixth People’s Hospital, Shanghai Jiaotong University School of Medicine, Shanghai 200025, China; 2 Key Laboratory for Advanced Materials and Joint International Research Laboratory of Precision Chemistry and Molecular Engineering, East China University of Science and Technology, Shanghai 200231, China; 3 Department of Imaging Medicine and Nuclear Medicine, Tongji Hospital, Shanghai 200065, China

**Keywords:** pancreatic cancer, chemotherapy, sonodynamic therapy, thermosensitive hydrogel

## Abstract

**Background:** Albumin-bound paclitaxel (nab-PTX) nanoparticles have been proven effective in treating advanced pancreatic cancer. However, the clinical application of nab-PTX nanoparticles is often associated with suboptimal outcomes and severe side effects due to its non-specific distribution and rapid clearance. This study aims to develop a novel nanoplatform that integrates sonodynamic therapy (SDT) and chemotherapy to enhance treatment efficacy and reduce systemic side effects. **Methods:** Bovine serum albumin (BSA) was conjugated with chlorin e6 and paclitaxel (PTX) to form stable nanoparticles (NPs). These NPs were then incorporated into a biodegradable poly(lactic-*co*-glycolic acid)–*b*-polyethylene glycol–*b*–poly(lactic-*co*-glycolic acid) hydrogel for targeted drug delivery. The system’s stability and drug release profile were analyzed, followed by in vitro studies to evaluate cellular uptake and cancer cell killing efficacy. In vivo evaluation was performed using pancreatic cancer xenograft models, with intratumoral injection of the drug-loaded hydrogel. **Results:** The developed hydrogel system demonstrated enhanced stability and sustained release of PTX. In vitro analyses revealed significant cellular uptake and synergistic cancer cell killing effects through combined SDT and chemotherapy. In vivo studies showed prolonged intratumoral retention of the drug and remarkable inhibition of tumor growth. **Conclusions:** This novel nanoplatform offers a promising approach for improving pancreatic cancer treatment by enhancing intratumoral drug retention and minimizing systemic side effects. The synergistic effects of SDT and chemotherapy demonstrate the potential of this strategy in achieving better therapeutic outcomes.

## 1. Introduction

Pancreatic cancer has high fatality rates, and early detection is challenging. In addition, chemotherapy is often required as a primary intervention for tumors that cannot be surgically removed [1,2,3]. The Food and Drug Administration (FDA) has approved albumin-bound paclitaxel (nab-PTX) NPs as a primary treatment for advanced pancreatic cancer, and nab-PTX NPs show high treatment efficacy [4,5]. However, in the clinical setting, conventional systemic chemotherapy often results in suboptimal outcomes and severe side effects because of the drugs’ non-selective distribution and quick elimination [6]. Therefore, innovative therapeutic approaches to enhance the effectiveness of pancreatic cancer chemotherapy while simultaneously mitigating systemic side effects are required.

In recent years, there has been growing interest in combination cancer therapies that synergistically enhance each treatment modality [7,8]. Among these, the use of a single nanoplatform integrating sonodynamic therapy (SDT) and chemotherapy has emerged as a promising strategy [8,9,10,11,12]. This approach aims to boost therapeutic efficacy, address drug resistance, and minimize undesirable side effects [13,14,15]. SDT, a noninvasive therapeutic modality, employs low-frequency, low-intensity ultrasound (US) to irradiate tumor areas where sonosensitizers have accumulated. This irradiation generates reactive oxygen species (ROS), thus triggering antitumor effects, including the direct killing and apoptosis of tumor cells [16,17,18]. Chlorin e6 (Ce6) is an FDA-approved second-generation photosensitizer and is also an effective and safe sonosensitizer for SDT [19,20,21]. However, challenges such as poor solubility leading to aggregation in aqueous solution, as well as issues with phototoxicity and low tumor accumulation after venous administration, must be addressed [22,23].

Localized drug administration at the tumor site by means of interventional radiology or within the resection cavity produced by surgery offers a promising and potentially superior alternative to venous administration, facilitating concentrated drug delivery to the tumor and reducing toxicity in healthy tissues [24,25,26,27,28]. An emerging trend in this domain is the use of biodegradable, thermosensitive, on-site developing hydrogels; the FDA-approved triblock copolymer poly(lactic-*co*-glycolic acid)–*b*-polyethylene glycol–*b*–poly(lactic-*co*-glycolic acid) (PLGA–PEG–PLGA), which is both biodegradable and biocompatible, has emerged as a flexible carrier suitable for delivering both hydrophobic and hydrophilic drugs [29,30]. A distinctive characteristic of PLGA–PEG–PLGA is its thermosensitive nature, enabling a transition from a liquid solution to a hydrogel (sol–gel) phase when exposed to temperature variations. Initially, at ambient temperature, the copolymer is a free-flowing liquid, simplifying the process of drug incorporation through straightforward mixing. Upon administration into the body, it responds to the physiological temperatures at the targeted site by transforming from liquid to a semi-solid gel. This transition is critical as the resultant gel serves as a sustained-release depot, methodically dispensing the encapsulated drug over an extended duration. This mechanism ensures a steady therapeutic effect, crucial for effective cancer treatment [31,32].

**Figure 1:**
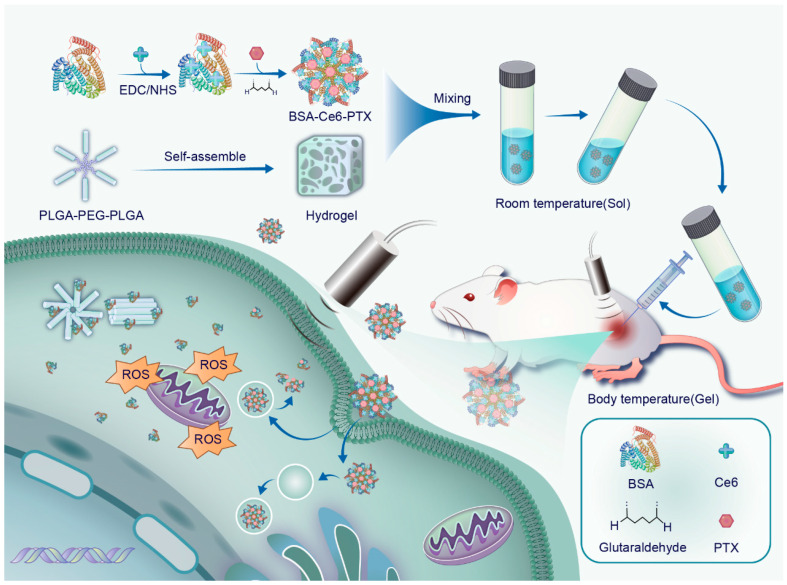
Schematic showing the preparation of the BCP/PLGA-PEG-PLGA hydrogel for synergistic SDT and chemotherapy.

Based on these considerations, we present an innovative bovine serum albumin (BSA)-based nanoparticle (NP) delivery system using an injectable thermosensitive hydrogel for the local delivery of BSA-Ce6-PTX (denoted as BCP) NPs, aimed at promoting synergistic combination therapy for pancreatic cancer. BSA was pre-modified with Ce6 (denoted as BC) and subsequently combined with PTX to form NPs. The BCPs were strategically integrated into a PLGA-PEG-PLGA hydrogel for targeted delivery to the tumor site. When administered as a solution, the thermosensitive hydrogel undergoes a phase transition to form a semisolid gel in response to body temperature, ensuring sustained NP release and the prolonged exposure of cancer cells to the drug. The combination of SDT and chemotherapy within this nanoplatform had a synergistic effect, enhancing therapeutic efficacy while minimizing systemic side effects. Further, in vitro experiments revealed a superior cancer-cell-killing efficacy, which was attributed to the sustained release of NPs. Further, in vivo studies on pancreatic cancer xenograft models demonstrated a significant inhibition of tumor growth with minimal systemic toxicity (Figure 1).

## 2. Results

**Figure 2:**
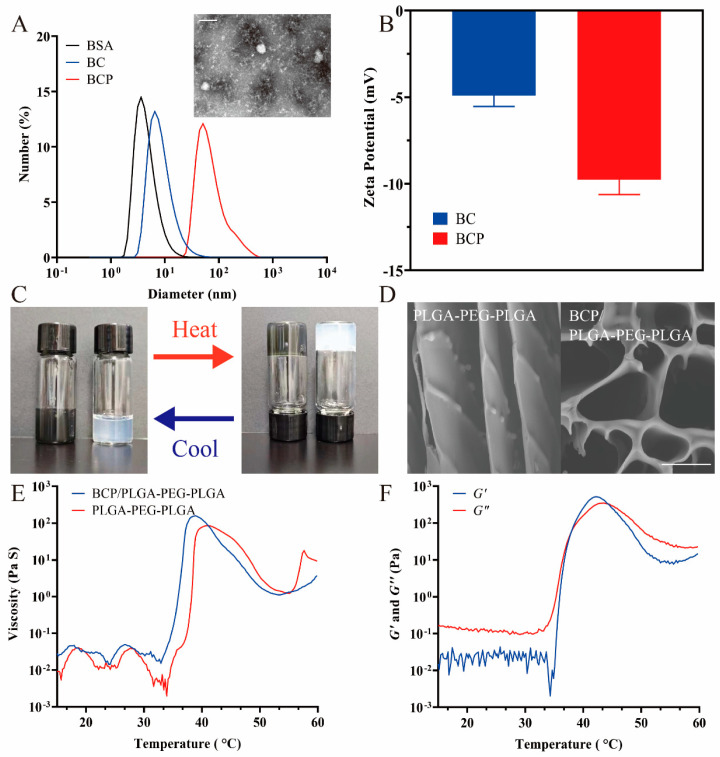
Characterization of NP hydrogel: (**A**) Hydrodynamic diameters of BSA, BC, BCP measured by DLS, and TEM image of BCP. (**B**) Zeta potentials of BC and BCP. (**C**) Observation of reversible sol–gel phase transition in BCP/PLGA-PEG-PLGA (**left**) and PLGA-PEG-PLGA (**right**) hydrogel across temperatures of 25 and 37 °C. (**D**) SEM image of hydrogels. (**E**) Examination of the viscosity changes in both the PLGA-PEG-PLGA and BCP/ PLGA-PEG-PLGA when subjected to heating. (**F**) Assessment of the variations in storage modulus (*G*’) and loss modulus (*G*”) for the BCP/PLGA-PEG-PLGA as temperature changes. Scale bar: 100 nm (**A**) and 5 μm (**D**).

**Figure A1:**
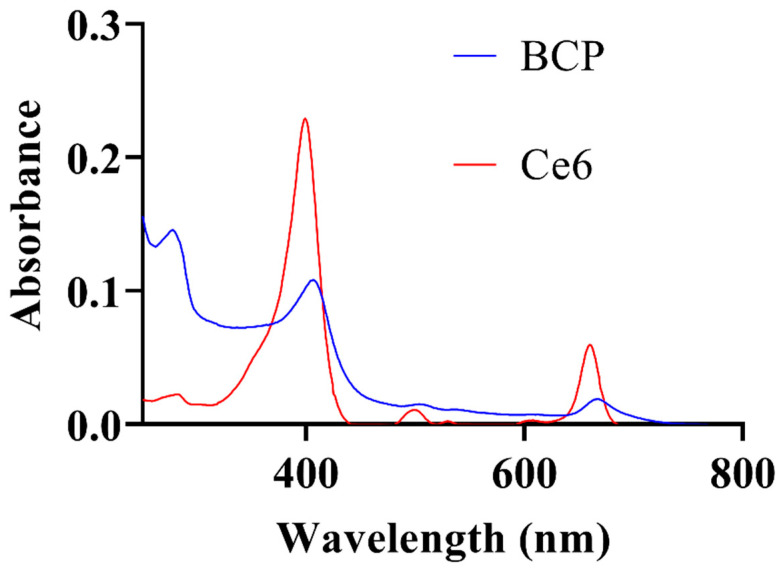
UV–vis–NIR absorption spectra of Ce6 and BCP.

TEM imaging revealed that BCP NPs have a spherical morphology and an average diameter of approximately 100 nm. This was confirmed by dynamic light scattering (DLS) measurements, which revealed the hydrodynamic size of the NPs to be 114.2 ± 3.2 nm (Figure 2A). The size discrepancy observed among BSA, BC, and BCP NPs is likely due to the protein aggregation triggered by PTX. Noteworthy is the observation that both BC and BCP exhibited negative surface charges, which almost doubled after nanoparticle formation (Figure 2B). Additionally, the NPs preserved the distinctive absorption peaks of Ce6 at 404 and 650 nm in their UV-vis–NIR absorption spectra (Figure A1). In addition, the drug loading efficiencies for Ce6 and PTX were substantial, reaching 36.11% for Ce6 and 29.43% for PTX, as validated through UV-vis absorbance spectra and HPLC analysis.

PLGA is a copolymer of lactic acid (LA) and glycolic acid (GA), with a ratio of LA:GA of 75:25. The PLGA1654(75:25)-PEG1500-PLGA1654(75:25) hydrogel, comprising a central PEG block flanked by PLGA blocks, displayed a reversible temperature-dependent sol–gel–sol transition attributed to micelle aggregation. The number-averaged molecular weight (*Mn*) of the copolymers, as determined by ^1^H NMR spectroscopy, was calculated to be 4808, with a PEG/PLGA ratio of 1500:3308. Usefully, the BCP NPs could be effectively integrated into the PLGA-PEG-PLGA hydrogel via a straightforward mixing process at room temperature. These hydrogels exhibited both sol and gel states, which were both responsive to temperature variations. Notably, both the BCP/PLGA-PEG-PLGA and pure PLGA-PEG-PLGA hydrogels maintained a non-flowing state for at least 1 min at 37 °C, indicating that gelation at physiological temperatures is possible (Figure 2C). SEM imaging unveiled a distinctive cross-linked surface network in the hydrogel composites, indicative of nanoparticle integration (Figure 2D).

**Figure A2:**
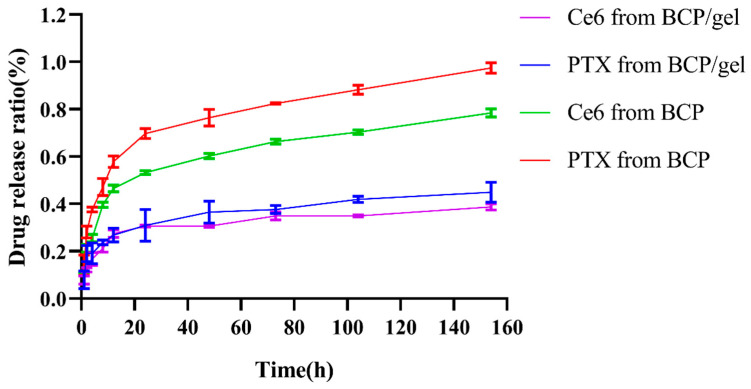
Drug release curve of PTX and Ce6 from BCP or BCP/gel.

The hydrogels’ thermosensitive transitions were further explored via rheological assessments. The rheological properties of the BCP/PLGA-PEG-PLGA and PLGA-PEG-PLGA composites were comparable, exhibiting similar viscosity changes upon gelation (Figure 2E). Below or at room temperature, BCP/PLGA-PEG-PLGA exhibited low storage (*G*’) and loss (*G*”) moduli, indicating high flowability and injectability. Heating to approximately 37 °C resulted in a significant rise (over three orders of magnitude) in both *G*’ and *G*”, signifying the creation of a cross-linked physical hydrogel (Figure 2F). In vitro drug release experiments under acidic conditions (PBS, pH 6.8) to mimic the tumor environment showed distinct cumulative release patterns for PTX and Ce6 (PBS, pH 6.8) revealed distinct cumulative release profiles of PTX and Ce6 (Figure A2). Within the initial 48 h, 75% of PTX was released for the BCP group and 36% was released for the BCP/PLGA-PEG-PLGA group, compared to 63% and 26% for Ce6, respectively. Both PTX and Ce6 exhibited sustained release even after 150 h in the BCP/PLGA-PEG-PLGA group. Specifically, PTX was released more rapidly than Ce6; this variance in release rates is ascribed to PTX’s non-covalent bonding. These findings suggest that the hydrogels’ three-dimensional framework effectively delays or inhibits premature drug release while enhancing drug concentration at tumor sites.

**Figure 3:**
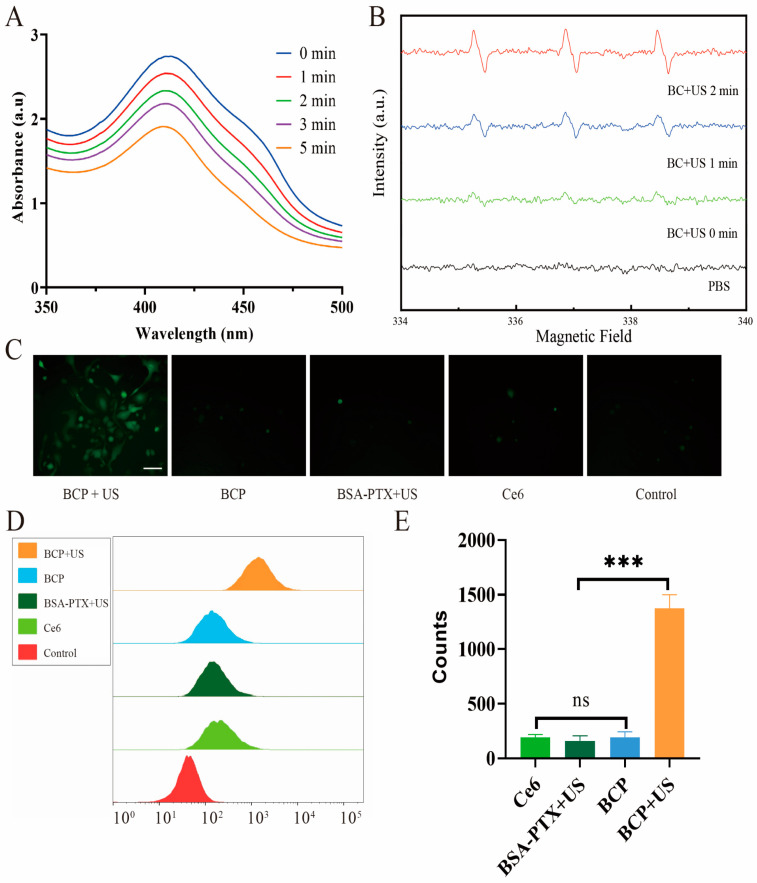
Sonodynamic and in vitro ROS generation efficacy. (**A**) Sonodynamic effect of Ce6. DPBF is oxidized by ^1^O_2_, resulting in a reduced absorbance intensity at 410 nm in the UV-vis absorbance spectra. (**B**) ESR spectra of BC irradiated by US for different periods. TEMP was used as the spin-trapping agent for ^1^O_2_ for detection by ESR. (**C**) Fluorescence images of ROS levels in PANC-1 cells stained with DCFH-DA after various treatments. (**D**,**E**) Quantitative analysis of fluorescent intensity of DCFH-DA determined by flow cytometry. Scale bar: 50 μm (**C**). *** *p* < 0.001 and ns: not significant by one-way ANOVA.

**Figure A3:**
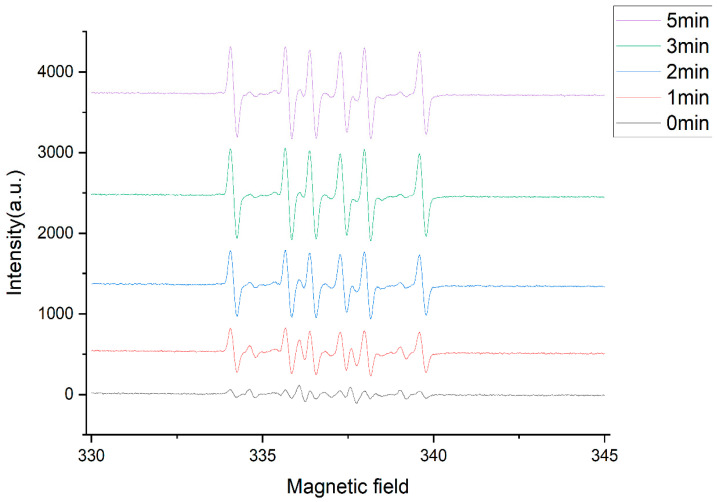
ESR spectra of BC irradiated by US for different periods. DMPO was used as the spin-trapping agent for ·OH for detection by ESR.

The generation of singlet oxygen (^1^O_2_) during SDT was examined using DPBF as a probe. DPBF reacts with ^1^O_2_, leading to a reduction in its absorbance at 410 nm in UV-vis absorbance spectra. A marked decrease in DPBF absorbance was noted following US irradiation in the presence of BCP (Figure 3A), signifying effective ^1^O_2_ production. For a more detailed investigation into the formation of ^1^O_2_ and hydroxyl radicals (·OH), ESR spectroscopy was utilized. Spin-trapping agents, specifically TEMP for ^1^O_2_ and DMPO for ·OH, were used to detect and visualize the signals associated with these ROS generated by the combination of US and BCP. The ESR spectra exhibited distinct signals characteristic of ^1^O_2_/·OH production when BCP was subjected to US (Figure 3B and Figure A3). To quantify the intracellular production of ROS in PANC-1 cells, 2′,7′-dichlorofluorescein diacetate (DCFH-DA), a commonly used ROS indicator, was employed. Notably, the highest DCF fluorescence signals, indicating increased ROS levels, were observed in cells treated with BCP and subsequent US irradiation. These findings were corroborated by fluorescence microscopy and flow cytometry (Figure 3C–E). The most intense DCF fluorescence signals were seen in cells pre-incubated with BCP and then treated with US. This underscores the potent ability of BCP to boost intracellular ROS production when activated by ultrasound during SDT.

**Figure 4:**
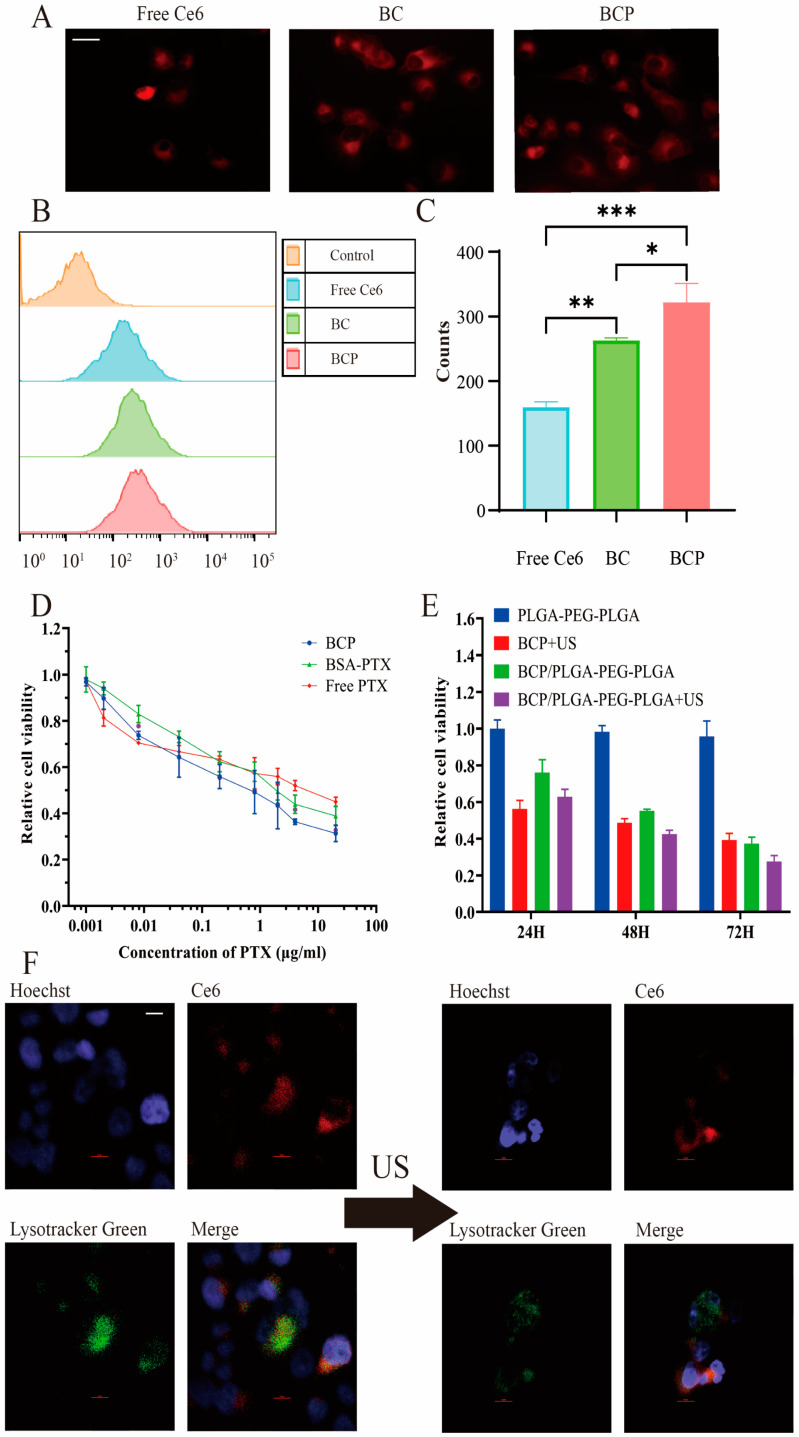
In vitro cell uptake and cytotoxicity experiments. (**A**) Observation of Ce6 fluorescence in PANC-1 cells following 4 h of incubation with free Ce6, BC, and BCP. (**B**,**C**) Flow cytometry data showing the accumulation of Ce6 in different groups. (**D**) Relative viabilities of PANC-1 cells after incubation with various concentrations of free PTX, BSA-PTX NPs, or BCP for 48 h. (**E**) Relative viabilities of PANC-1 cells after incubation with PLGA-PEG-PLGA, BCP, and BCP/PLGA-PEG-PLGA with or without US irradiation. (**F**) Confocal fluorescence microscopy was used to image PANC-1 cells before and after US exposure, incubated with BCP NPs. In these images, green represents LysoTracker-stained endosomes/lysosomes, red indicates Ce6 fluorescence, and blue marks the Hoechst-stained cell nuclei. Scale bar: 25 μm (**A**) and 10 μm (**F**). * *p* < 0.05, ** *p* < 0.01 and *** *p* < 0.001 by one-way ANOVA.

**Figure A4:**
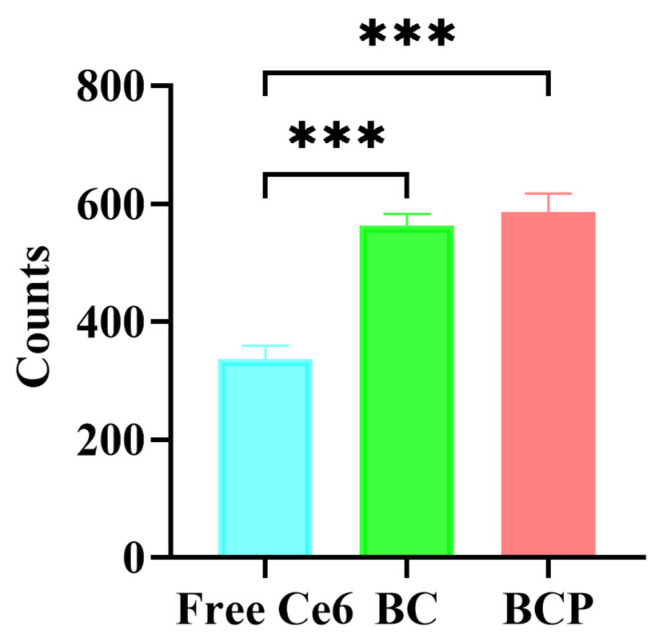
Flow cytometry data showing the accumulation of Ce6 in different groups irradiated by US. *** *p* < 0.001 by one-way ANOVA.

The cellular uptake efficiency of BCP NPs was analyzed in PANC-1 cells. Cells were incubated with Ce6, BC, or BCP for 2 h at 37 °C and then washed with PBS to eliminate extracellular NPs. Fluorescence microscopy and flow cytometry results demonstrated significant Ce6 fluorescence in cells treated with BCP (as shown in Figure 4A–C), confirming effective cellular uptake. In comparison, cells exposed to free Ce6 and BC exhibited weaker fluorescence signals. The influence of US irradiation on cellular membrane permeability and subsequent NP uptake was also examined. Post-incubation with the NPs, cells were subjected to US irradiation. Flow cytometry analysis revealed an almost twofold increase in fluorescence intensity in US-treated groups, indicating enhanced NP internalization (Figure A4). This confirms the efficient cellular uptake of BCP, facilitated by their size and BSA coating, with US further improving their delivery into tumor cells.

The cytotoxic effects of various PTX formulations were also evaluated in vitro on PANC-1 cells treated with free PTX (in DMSO), BSA-PTX, or BCP for 48 h. Cell viability was assessed using the MTT assay. The cytotoxicity of the BCP formulation was found to be superior to that of free PTX and BSA-PTX (Figure 4D), likely due to BCP’s increased cellular uptake efficiency.

**Figure A5:**
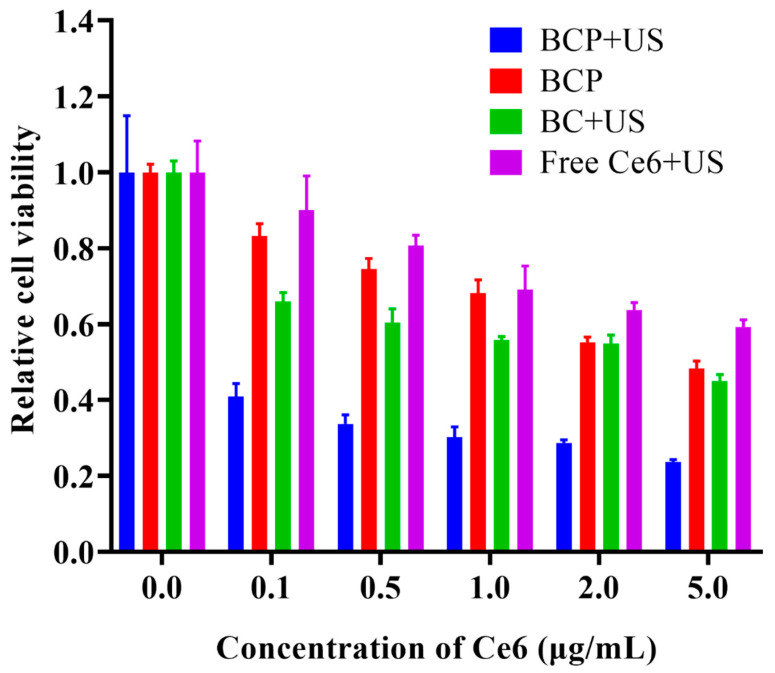
Relative viabilities of PANC-1 cells after incubation with free Ce6, BC, and BCP with or without US irradiation.

For evaluating the efficacy of SDT, various Ce6 formulations were tested on PANC-1 cells with or without US irradiation. After 4 h of incubation, cells were exposed to US (1 MHz, 1 W/cm^2^, for 2 min), followed by an additional 24 h of incubation. Cell viability was again determined using the MTT assay. A pronounced synergistic effect from combined SDT and chemotherapy was observed in BCP NPs-treated cells post-US irradiation, showing enhanced cancer cell killing efficacy (Figure A5).

**Figure A6:**
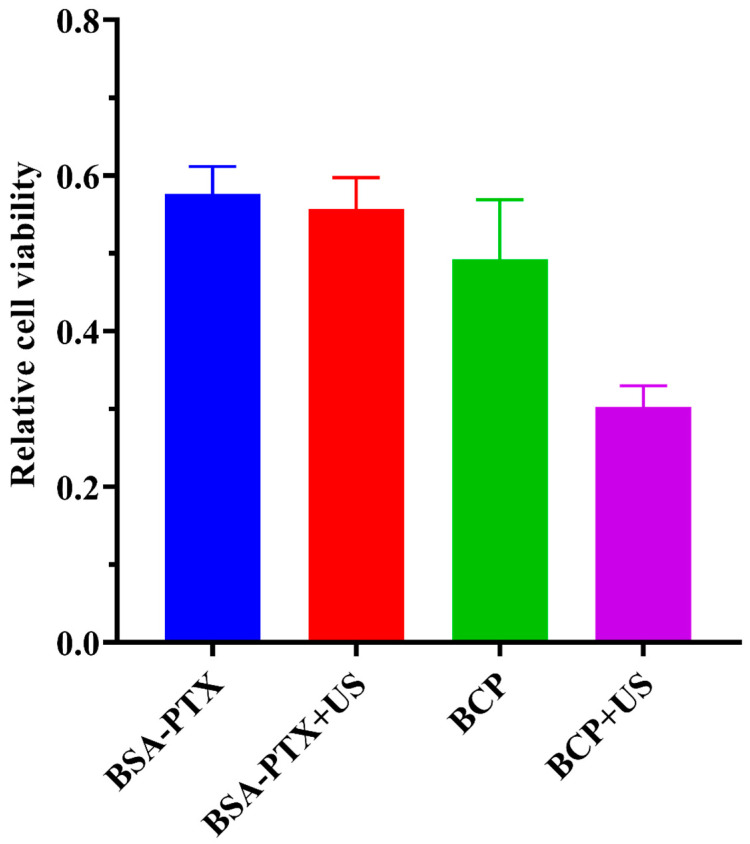
Relative viabilities of PANC-1 cells after incubation with BSA-PTX and BCP with or without US irradiation.

To confirm that the results were due to Ce6 and not just US, PANC-1 cells were treated with BSA-PTX or BCP with or without US irradiation. After 48 h of incubation, cell viability was assessed via the MTT assay. Compared to BSA-PTX alone, BSA-PTX+US showed a minor reduction in cell activity, attributed to improved nanoparticle distribution facilitated by US. However, BCP+US demonstrated a significant decrease in cell viability, indicating substantial damage to tumor cells by ROS generated from Ce6 under US irradiation (Figure A6).

The drug-loaded PLGA-PEG-PLGA hydrogel system’s cytotoxicity was assessed over various incubation periods using the MTT assay. A Transwell co-culture system simulated a drug storage and release environment, with medium replacement every 24 h. The viability of PANC-1 cells was measured after 24, 48, and 72 h of incubation with free PLGA-PEG-PLGA, BCP, and BCP/PLGA-PEG-PLGA (Figure 4E). During the initial period, US irradiation was applied to the BCP and BCP/PLGA-PEG-PLGA groups. The PLGA-PEG-PLGA hydrogel exhibited minimal cytotoxicity, confirming its biocompatibility. BCP/PLGA-PEG-PLGA+US initially showed lower cytotoxicity than BCP+US, likely due to delayed drug release. Over time, a decrease in cell viability was noted, especially in the BCP/PLGA-PEG-PLGA+US group, indicating sustained drug release and a significant synergistic therapeutic effect.

To understand the mechanism behind this synergistic effect, confocal fluorescence microscopy was conducted before and after US irradiation (Figure 4F). Prior to US, Ce6 fluorescence significantly co-localized with endo/lysosomes, marked by LysoTracker (green). Following US treatment, there was a noticeable decrease in LysoTracker fluorescence and a broader distribution of Ce6 fluorescence within the cytoplasm. This implies that the sonodynamic effect facilitates the degradation of endo/lysosomes and assists in the endosomal release of NPs.

**Figure 5:**
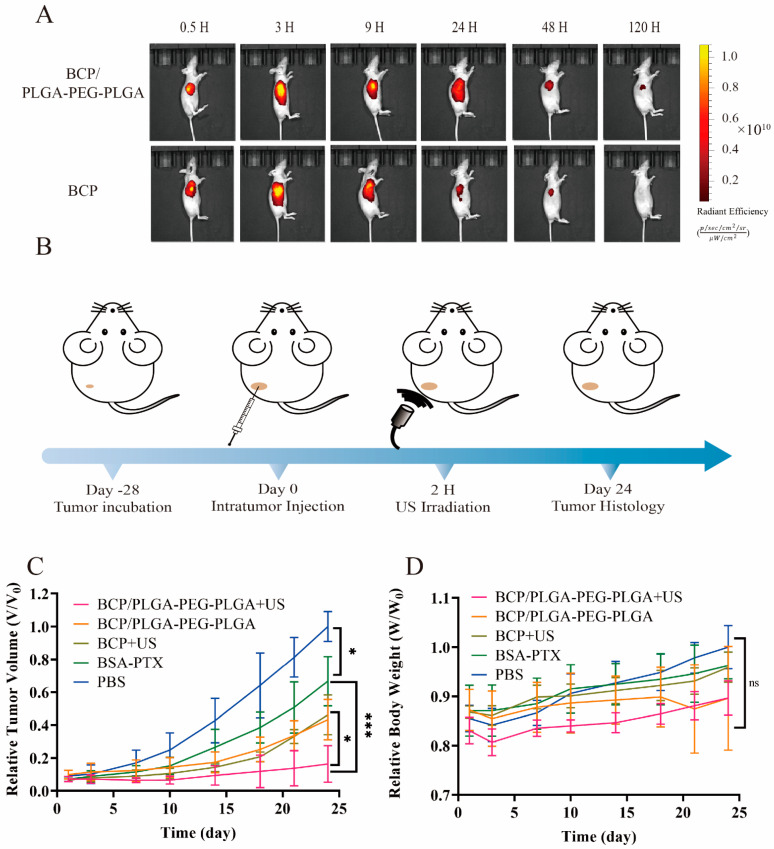
In vivo fluorescence imaging and evaluation of antitumor efficacy. (**A**) Real-time intravital images showcasing the retention of Ce6 within the tumor. (**B**) Schematic representation of evaluating antitumor efficacy through treatment of tumors with NPs/hydrogels and US irradiation. (**C**) Tumor volume changes over time. (**D**) Changes in the body weight of mice over time. Every data point indicates the average value ± standard deviation (S.D.), with a sample size of four mice (n = 4). * *p* < 0.05, *** *p* < 0.001 and ns: not significant by one-way ANOVA.

An in vivo imaging experiment utilized a subdermal pancreatic cancer graft model in nude mice to evaluate the sustained release properties of the PLGA-PEG-PLGA hydrogel. BCP and BCP/PLGA-PEG-PLGA were administered locally to tumors, and the intratumoral persistence of Ce6 was monitored in real-time using an IVIS imaging system. (Figure 5A). The fluorescence intensity from BCP diminished significantly within 48 h, reflecting rapid in vivo clearance. Conversely, the group injected with BCP/PLGA-PEG-PLGA exhibited prolonged and steady Ce6 dispersion within the tumor area for more than 120 h. This underscores the effectiveness of the PLGA-PEG-PLGA hydrogel as a system for direct tumor-targeted drug delivery, guaranteeing increased drug concentrations, specifically at the tumor location, and regulated drug dissemination.

**Figure 6:**
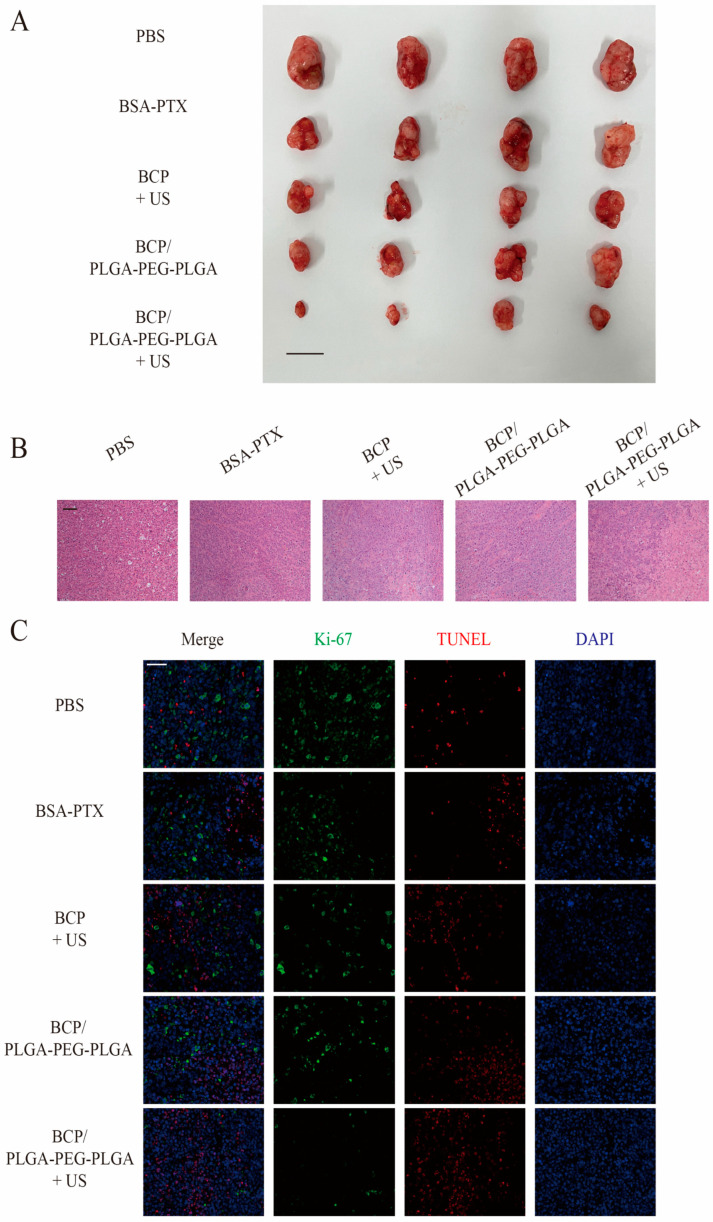
Tumors and histological images. (**A**) Photographs of tumors extracted from mice in different groups on the final day of treatments (day 24). (**B**,**C**) Histological sections of PANC-1 xenografts from different treatment groups stained with H&E and dual-stained for Ki-67, TUNEL and DAPI. Scale bar: 1 cm (**A**), 100 μm (**B**,**C**).

Inspired by the successful in vitro cellular uptake, therapeutic effectiveness, and favorable in vivo retention, combination therapy in pancreatic cancer xenograft models was evaluated. Mice with tumors were treated with a single dose, either intratumorally (i.t.) or intravenously (i.v.), as depicted in Figure 5B. Subsequent changes in tumor volume were monitored over 24 days (Figure 5C). Tumor size escalated rapidly in the PBS-treated group. Following i.v. administration of BSA-PTX, tumor growth was modestly inhibited, likely due to limited drug accumulation within the tumor. Local injection of BCP with US irradiation initially impeded tumor growth, but this was followed by rapid tumor progression, likely due to swift drug clearance. Remarkably, intratumoral injection of the BCP/PLGA-PEG-PLGA composites into the tumor notably hindered tumor expansion compared to BSA-PTX, demonstrating sustained drug release within the tumor region. Additionally, mice receiving BCP/PLGA-PEG-PLGA combined with US treatment exhibited the furthest reduced tumor growth rate, leading to the smallest tumors at the end of the 24 days (Figure 6A). These findings indicate the promise of the thermosensitive hydrogel system as a vehicle for extended drug delivery to specific tumor areas, with the addition of sonodynamic therapy enhancing the therapeutic impact.

Furthermore, alterations in the body weight of the mice were recorded to assess systemic toxicity in reaction to various treatment methods (Figure 5D). Compared to the PBS control group, mice treated with either BCP or BCP/PLGA-PEG-PLGA showed no significant changes in body weight or clinical indications, affirming the safety and appropriateness of these formulations for intratumoral administration. 

H&E staining and Ki-67/TUNEL dual-labeling experiments were conducted to evaluate morphological integrity and to assess the effects on cell proliferation and apoptosis across different treatment groups. H&E staining revealed normal cell structures and densities in the PBS control group (Figure 6B). The BSA-PTX and BCP/PLGA-PEG-PLGA groups displayed no significant morphological changes compared to the control, likely due to the limited effect of chemotherapy alone. In contrast, the BCP ultrasound and BCP/PLGA-PEG-PLGA ultrasound groups showed looser tissue structures and reduced cell densities, indicating increased cell death.

In Ki-67 and TUNEL dual staining (Figure 6C), baseline levels of proliferation and apoptosis matched untreated tumor cell behavior. Ki-67 expression was slightly reduced in the BSA-PTX group, reflecting PTX’s antiproliferative effect. The BCP/PLGA-PEG-PLGA group showed further Ki-67 reduction, likely due to the sustained release from the hydrogel system. In the BCP ultrasound and BCP/PLGA-PEG-PLGA ultrasound groups, significant Ki-67 reduction with increased TUNEL staining suggested that sonodynamic therapy with chemotherapy effectively inhibited proliferation and induced apoptosis, with the hydrogel enabling more stable drug release (Figure 5D).

**Figure A7:**
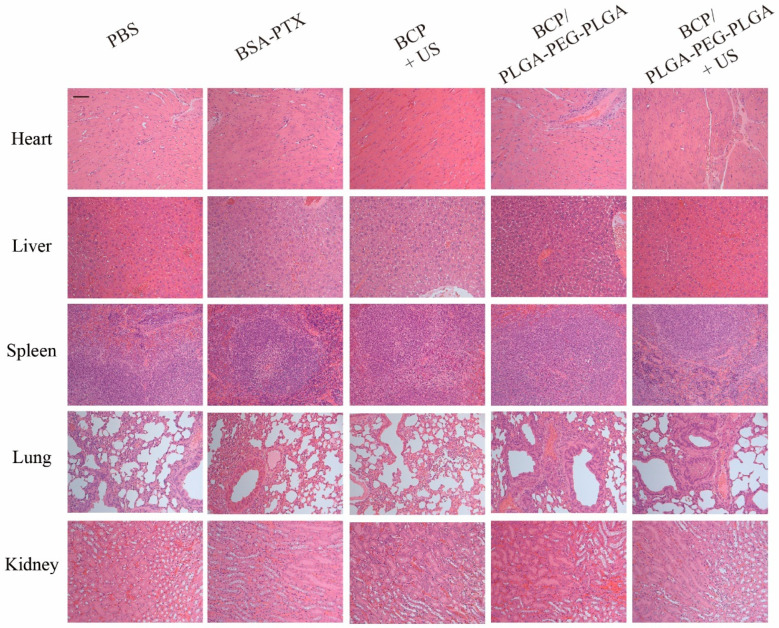
Histological images, with H&E staining, of key organs from tumor-bearing mice following different treatments at day 24. Scale bar: 100 μm.

Histopathological examination of critical organs (heart, liver, spleen, lungs, and kidneys) was also performed on day 24 post-treatment. Tissue sections from mice treated with the BCP/PLGA-PEG-PLGA displayed histological characteristics akin to those treated with PBS (Figure A7), implying that localized delivery of BCP/PLGA-PEG-PLGA is associated with minimal systemic toxicity.

## 3. Discussion

Our study presents a novel approach to addressing the challenges in pancreatic cancer treatment by combining albumin-based nanoparticles (BCP) with a thermosensitive PLGA-PEG-PLGA hydrogel for localized drug delivery. The findings highlight the therapeutic potential of integrating SDT and chemotherapy into a single nanoplatform, demonstrating significant tumor inhibition and reduced systemic side effects.

The results demonstrate the synergistic effect of SDT and chemotherapy, where Ce6, acting as a sonosensitizer, effectively generates ROS under US irradiation. This ROS production amplifies the cytotoxicity of PTX, leading to enhanced cancer cell apoptosis. The hydrogel’s sustained release properties ensured prolonged intratumoral drug retention, significantly inhibiting tumor growth in vivo while minimizing systemic toxicity. Compared with conventional systemic chemotherapy, which often suffers from rapid clearance and widespread toxicity [6], this nanoplatform achieves superior tumor drug accumulation and therapeutic efficacy.

In comparison to existing monotherapies or standard chemotherapy regimens [1], this integrated system provides a dual-mode mechanism that effectively overcomes the limitations of rapid drug elimination and insufficient tumor targeting. The localized application of PLGA-PEG-PLGA hydrogel, combined with US-triggered SDT, represents a significant improvement over previous nanoparticle systems, as it offers sustained and controlled drug release. These findings align with and extend recent advancements in sonodynamic therapy and hydrogel-based drug delivery platforms [33].

Mechanistically, US irradiation facilitates the endosomal escape of Ce6-loaded nanoparticles, as evidenced by fluorescence imaging. This process enhances intracellular drug availability, boosting the overall therapeutic impact. Furthermore, the ROS generated by Ce6 under US irradiation induces oxidative damage, which complements the cytotoxic effects of PTX. This dual action underscores the rationale for combining SDT and chemotherapy within a single nanoplatform.

One of the study’s key strengths is its use of FDA-approved components, such as Ce6 and PLGA-PEG-PLGA hydrogel, enhancing the translational potential of this approach. Additionally, the comprehensive evaluation, including in vitro, in vivo, and mechanistic studies, strengthens the reliability of the findings.

Nevertheless, our study has certain limitations. The use of intratumoral injection, while effective for localized delivery, is invasive and may limit clinical applicability. However, interventional medicine offers a promising solution by enabling precise and minimally invasive drug delivery. Through advanced imaging guidance, interventional techniques can ensure accurate hydrogel placement within tumors, potentially bridging the gap between efficacy and clinical feasibility [34]. Furthermore, the study did not explore potential immune responses or the long-term biocompatibility of the hydrogel system. The efficacy of the system against metastases or in combination with immune therapies also remains unexplored and warrants further investigation. Future studies should focus on optimizing the system for non-invasive delivery methods, such as ultrasound-guided systemic targeting, to improve clinical feasibility. Additionally, exploring the platform’s compatibility with other therapeutic agents, including immune checkpoint inhibitors or anti-angiogenic drugs, could enhance its efficacy. Long-term studies evaluating tumor recurrence and overall survival in animal models will provide further insights into the durability and translational potential of this approach.

In conclusion, our study offers a promising therapeutic strategy for pancreatic cancer by integrating SDT and chemotherapy within a biodegradable, thermosensitive hydrogel system. The synergistic effects, coupled with localized drug delivery and reduced systemic toxicity, represent a significant advancement in nanoplatform-based cancer therapies. With further refinement and validation, this approach holds substantial potential for clinical translation, offering a new avenue for improving outcomes in pancreatic cancer treatment.

## 4. Materials and Methods

### 4.1. Material

Bovine Serum Albumin (BSA), 1-ethyl-3-(3-dimethylaminopropyl)carbodiimide (EDC), N-hydroxysuccinimide (NHS), Ce6, methanol, dimethyl sulfoxide (DMSO), 25% glutaraldehyde, phosphate-buffered saline (PBS), Tween-80, Dichloromethane (DCM), and PLGA-PEG-PLGA hydrogel were obtained from Tanshtech (Tanshui Technology Co., Ltd., Guangzhou, China).

### 4.2. Preparation and Characterization of BCP NPs

#### 4.2.1. Synthesis of BC NPs

Ce6 was initially dispersed in DMSO (200 µL). Then, a mixture of Ce6, EDC, and NHS was stirred in darkness for 2 h at room temperature to synthesize a Ce6–NHS complex. This complex was subsequently incorporated into the BSA solution in PBS. 

The mixture was stirred overnight in the dark. To purify BC, the mixture was centrifuged at 14,800 rpm for 5 min to remove aggregates, followed by three cycles of ultrafiltration (molecular weight cut-off (MWCO): 10 kDa) to isolate free Ce6.

#### 4.2.2. Synthesis of BCP NPs

BC (20 mg) was dissolved in PBS (10 mL) with constant stirring. Then, PTX solution (200 µL, 10 mg/mL in methanol) was incrementally introduced. The formation of an emulsion was observed, and 25% glutaraldehyde (10 µL) was added. The mixture was then left to stir throughout the night at room temperature. Excess PTX was removed by centrifugation at 5000 rpm for 5 min. Ultrafiltration centrifugation (MWCO = 100 kDa) was used to remove the residual BSA, methanol, and glutaraldehyde.

#### 4.2.3. Synthesis of BSA-PTX NPs

The methodology for synthesizing BSA-PTX NPs was analogous to that for BCP, but BC was substituted with BSA.

#### 4.2.4. Characterization of BCP

The morphology and structure of BCP NPs stained with 1 wt.% phosphotungstic acid were observed by transmission electron microscopy (TEM; FEI Tecnai F20). 

Absorbance spectra in the UV-vis-NIR range were captured with a PerkinElmer Lambda 750 spectrophotometer. 

The hydrodynamic diameters and zeta potential of the samples were measured using a Zetasizer Nano-ZS (Malvern Instruments, Malvern, UK). 

To quantify singlet oxygen (^1^O_2_) and hydroxyl radicals (·OH), an electron spin resonance (ESR) spectrometer (Bruker EMXplus) was employed using 2,2,6,6-tetramethylpiperidine (TEMP) and dimethylpyridine N-oxide (DMPO) as spin-trapping agents. The presence of ^1^O_2_ was confirmed using 1,3-diphenyliso-benzofuran (DPBF). Subsequently, the sample solutions were treated with US at a power density of 1 W/cm^2^ and a frequency of 1 MHz for varying periods (0–5 min). The reduction in the fluorescence intensity of DPBF was measured at a wavelength of 410 nm.

### 4.3. Preparation and Sol–Gel Phase Transition Properties of BCP/PLGA-PEG-PLGA

#### 4.3.1. Preparation of PLGA-PEG-PLGA and BCP/PLGA-PEG-PLGA

First, PLGA-PEG-PLGA (1 mg) was dissolved in PBS (4 mL) at pH 7.4 and room temperature and stirred continuously until it was completely dissolved. The resulting solution had a PLGA-PEG-PLGA concentration of 25%. After sterilization through a 0.22 μm filter membrane, BCP NPs were integrated into the PLGA-PEG-PLGA hydrogel. The hydrogel–NP mixture was preserved at 4 °C for later application.

#### 4.3.2. Phase Diagram Assessment

The thermosensitive sol–gel transition of the BCP/PLGA-PEG-PLGA hydrogel was evaluated using the test tube inversion method. A 4 mL aliquot of the hydrogel was gradually heated from 25 °C, with observations of the sol-to-gel phase change being made. This transition was noted when no substantial flow occurred upon inverting the vial for 1 min. Moreover, the phase transition characteristics of the PLGA-PEG-PLGA hydrogel in isolation were also recorded.

#### 4.3.3. Characterization of BCP/PLGA-PEG-PLGA

Scanning electron microscopy (SEM) of the hydrogels was performed using a Regulus8220 instrument. The dynamic rheological properties of BCP/PLGA-PEG-PLGA (25 wt.%) and PLGA-PEG-PLGA (25 wt.%) were analyzed using a HAAKE MARS 60 rheometer equipped with parallel plates (diameter: 35 mm). The samples were subjected to temperature variations from 10 to 60 °C at a heating rate of 1 °C/min, and the storage modulus (*G*’), loss modulus (*G*”), and viscosity were recorded at a frequency of 1 Hz.

### 4.4. Drug Release

The in vitro release kinetics of PTX and Ce6 from the hydrogel were assessed using a 24-well Transwell system. Each lower chamber was filled with PBS (1 mL) containing 1% Tween-80. The upper inserts were loaded with either BCP/PLGA-PEG-PLGA (25 wt.%) or BCP (200 μL). The inserts were subsequently kept at 37 °C for 15 min to facilitate the formation of the hydrogel. The entire assembly was incubated at 37 °C with gentle shaking at 60 rpm. The release medium was collected and replaced with prewarmed PBS containing 1% Tween-80 at predetermined intervals. The released Ce6 was quantified using a UV-Vis–NIR spectrophotometer. For PTX quantification, the drug was extracted using dichloromethane (DCM) and analyzed using high-performance liquid chromatography (HPLC) at 227 nm. The mobile phase was administered at a flow rate of 1 mL/min.

### 4.5. Cell Experiments

#### 4.5.1. Cell Culture

PANC-1 cells were obtained from the National Collection of Authenticated Cell Cultures and cultured in Dulbecco’s modified Eagle’s medium (DMEM) supplemented with 10% fetal bovine serum (FBS), 1% penicillin, and streptomycin. The cells were maintained in a humidified incubator at 37 °C with 5% CO_2_.

#### 4.5.2. Cellular Uptake

To examine the cellular uptake of BCP NPs with or without US irradiation, PANC-1 cells were seeded in 12-well plates and exposed to free Ce6, BC, and BCP at a concentration of 10 µg/mL (n.b., this is the Ce6 equivalent concentration). Following this, the cells were either treated with US (1.0 MHz, 1 W/cm^2^, 2 min) or left untreated, followed by incubation for an additional 2 h. Subsequently, cells were washed with PBS for fluorescence microscopy and flow cytometry measurements.

#### 4.5.3. Intracellular Reactive Oxygen Species

To assess the levels of reactive oxygen species (ROS) in cells, PANC-1 cells were seeded in 12-well plates and treated with free Ce6, BSA-PTX, and BCP with or without US at a concentration of 10 µg/mL (Ce6 equivalent concentration) for 4 h. Following this, the cells were washed with PBS, and 2′,7′-dichlorofluorescein diacetate (DCFH-DA) was added for an additional 4 h. After US irradiation, flow cytometry and fluorescence microscopy experiments were carried out, and the DCF fluorescence signals were measured.

#### 4.5.4. In Vitro Cytotoxicity

To evaluate the cytotoxic effects of various PTX formulations, PANC-1 cells were cultivated in 96-well plates and then treated with different concentrations of free PTX, BSA-PTX NPs, or BCP NPs for a duration of 48 h. Cell survival rates, compared to those of the untreated controls, were determined using a standard thiazolyl tetrazolium (MTT) assay.

For SDT, PANC-1 cells in 96-well plates were combined with varying amounts of free Ce6, BC, or BCP NPs. Following a 4 h incubation period, these cells underwent US irradiation (1.0 MHz, 1 W/cm^2^, 2 min). The culture medium was then replaced with a fresh one, and the cells underwent an additional 24 h of incubation. Similarly, PANC-1 cells were treated with BSA-PTX, or BCP at a PTX-equivalent concentration of 0.8 µg/mL. After 4 h of incubation, these cells were either exposed to US (1.0 MHz, 1 W/cm^2^, 2 min) or left untreated. Post-irradiation, the culture medium was refreshed, and the cells were incubated for another 48 h. Cell viability was then assessed using a standard MTT assay.

Regarding combination therapy, PANC-1 cells were seeded in a 96-well Transwell (Corning) co-culture system and mixed with different concentrations of PLGA-PEG-PLGA, BCP, or BCP/PLGA-PEG-PLGA. In this process, 200 µL of the PANC-1 cell suspension was added to the lower chambers, while 100 µL of PLGA-PEG-PLGA, BCP, or BCP/PLGA-PEG-PLGA solutions was introduced into the upper inserts. After 4 h of incubation, groups treated with BCP and BCP/PLGA-PEG-PLGA were exposed to US (1.0 MHz, 1 W/cm^2^, 2 min). The medium in all groups was renewed every 24 h. Cell viability was then determined using the MTT assay at 24, 48, and 72 h post-incubation.

#### 4.5.5. Organelle Localization

To explore the underlying processes of combined SDT and chemotherapy, PANC-1 cells underwent treatment with BCP for 4 h. Post-treatment, these cells were washed with PBS to eliminate any residual nanoparticles. This was followed by a 5 min ultrasonic irradiation session (1.0 MHz, 1 W/cm^2^, 2 min). Subsequently, the cells were maintained at 37 °C for an additional 4 h. Prior to confocal microscopy, specific organelle labeling was conducted: LysoTracker (green) was used to highlight endosomes/lysosomes, and Hoechst (blue) was applied to visualize the nuclei.

### 4.6. In Vivo Antitumor Experiments

#### 4.6.1. Pancreatic Cancer Xenograft Models

BALB/c nude mice (female, 20 ± 2 g) were purchased from Gempharmatech Co., Ltd. (Nanjing, China). Mice were kept in a pathogen-free (SPF) environment with unrestricted access to both food and water. All procedures involving animals were performed in compliance with ethical standards and received approval from the relevant institutional review board. To establish pancreatic cancer xenografts, 1 × 10^6^ PANC-1 cells suspended in 50 μL of a matrigel were subcutaneously injected into the dorsal region of each mouse. The mice were included in the study once their tumor volumes reached between 100 and 150 mm^3^.

#### 4.6.2. In Vivo Imaging

For the purpose of conducting in vivo imaging studies, a volume of 100 μL containing either BCP or BCP/PLGA-PEG-PLGA (with a Ce6 equivalent concentration of 10 μg/mL) was injected directly into the tumors. Imaging was carried out utilizing an in vivo imaging system (IVIS; Lumina III, Perkin Elmer, Caliper Life Sciences, Hopkinton, MA, USA). During the imaging process, the mice were under anesthesia, and fluorescence images were captured at specific time intervals, 0.5, 3, 9, 24, 48, and 120 h, post-injection. The analysis of these images was performed using the integrated software (Living Image Version: 4.7.4.21053) of the imaging system.

#### 4.6.3. In Vivo Anti-Tumor Efficiency

Nude mice with subcutaneous PANC-1 tumors were allocated into five distinct groups, each comprising four animals: (a) a control group receiving PBS, (b) a group receiving BSA-PTX administered intravenously (i.v.), (c) a group receiving BCP with intratumoral ultrasound (US) application (i.t.), (d) a group with BCP/PLGA-PEG-PLGA administered i.t., and (e) a group administered BCP/PLGA-PEG-PLGA combined with US treatment i.t. The dosages of Ce6 and PTX were standardized at 0.3 and 0.2 mg/mL, respectively. US was applied to the relevant groups at a frequency of 1.0 MHz and a power density of 1 W/cm^2^ for 2 min, 24 h after the injection. Tumor dimensions were measured twice weekly using digital calipers to record length and width. Tumor volumes were calculated using the formula width^2^ × length/2. Comparative tumor volumes and body weights were assessed against the PBS group values noted on day 24. On the 24th day following treatment, the mice were humanely euthanized. The tumors and key organs were then extracted, weighed, preserved in 4% PBS-buffered paraformaldehyde, and subsequently embedded in paraffin wax for additional analysis.

#### 4.6.4. Histology

Tumor tissues and vital organs (including the heart, liver, spleen, lungs, and kidneys) were fixed in 4% paraformaldehyde, subjected to a graded ethanol dehydration process, and then embedded in paraffin. Tissue sections, each 5 μm thick, were prepared and stained with hematoxylin and eosin (H&E) for microscopic histopathological evaluation.

### 4.7. Statistical Analysis

Statistical analysis of the data was conducted using R software (version 4.0.1). The data are displayed as mean values ± standard deviation (S.D.). Group comparisons were performed using a one-way ANOVA. A *p*-value less than 0.05 was considered to indicate statistical significance.

## 5. Conclusions

We present a compelling strategy for the treatment of advanced pancreatic cancer using innovative albumin-based NPs (BCP NPs) and a biodegradable PLGA-PEG-PLGA hydrogel system. BCP, which is designed to achieve targeted drug delivery, effectively addresses the challenges associated with pancreatic cancer treatment, including the inherent poor solubility of Ce6 and rapid clearance of therapeutic agents. The combination of SDT and chemotherapy within a single nanoplatform resulted in a synergistic effect, enhancing therapeutic efficacy while mitigating systemic side effects. Further, the integration of Ce6 as a sonosensitizer proved to be a crucial element that contributed to enhanced intracellular ROS production and reinforced the therapeutic impact of SDT. In addition, the sustained release of NPs from the thermosensitive hydrogel ensured prolonged exposure to cancer cells, yielding a significant inhibition of tumor growth in in vivo experiments. Importantly, localized administration of the drug-loaded hydrogel minimized systemic toxicity, as evidenced by stable body weights and the histological assessment of vital organs. Overall, the proposed combination therapy, facilitated by novel albumin-based NPs and a PLGA-PEG-PLGA hydrogel, represents a promising advancement in pancreatic cancer treatment, offering a balance between therapeutic effectiveness and reduced systemic impact. Further exploration and clinical translation of this innovative approach has the potential to improve the outcomes of patients with pancreatic cancer.

## Data Availability

Data are contained within the article.
